# Safety of celecoxib and nonselective nonsteroidal anti-inflammatory drugs in juvenile idiopathic arthritis: results of the phase 4 registry

**DOI:** 10.1186/1546-0096-12-29

**Published:** 2014-07-16

**Authors:** Rachel E Sobel, Daniel J Lovell, Hermine I Brunner, Jennifer E Weiss, Paula W Morris, Beth S Gottlieb, Elizabeth C Chalom, Lawrence K Jung, Karen B Onel, Lisa Petiniot, Donald P Goldsmith, Kabita Nanda, Michael Shishov, Staci Abramsky, James P Young, Edward H Giannini

**Affiliations:** 1 Pfizer Inc, 235 East 42nd St, MS#219-9-1, New York, NY 10017, USA; 2 Cincinnati Children’s Hospital Medical Center, Cincinnati, OH, USA; 3 Hackensack University Medical Center, Hackensack, NJ, USA; 4 University of Arkansas for Medical Science, Little Rock, AR, USA; 5 Cohen Children’s Medical Center of New York, New Hyde Park, NY, USA; 6 St. Barnabas Medical Center, Livingston, NJ, USA; 7 Children’s National Medical Center, Washington, DC, USA; 8 University of Chicago, Chicago, IL, USA; 9 Specially for Children, Dell Children’s Medical Center, Austin, TX, USA; 10 St. Christopher’s Hospital for Children/Drexel College of Medicine, Philadelphia, PA, USA; 11 Rainbow Babies & Children’s Hospital, Cleveland, OH, USA; 12 Current address: Seattle Children’s Hospital, Seattle, WA, USA; 13 Phoenix Children’s Hospital, Phoenix, AZ, USA; 14 United BioSource Corporation, Ann Arbor, MI, USA

**Keywords:** Celecoxib, Juvenile idiopathic arthritis, NSAIDs, Safety

## Abstract

**Background:**

This study aimed to assess long-term safety and developmental data on juvenile idiopathic arthritis (JIA) patients treated in routine clinical practice with celecoxib or nonselective nonsteroidal anti-inflammatory drugs (nsNSAIDs).

**Methods:**

Children aged ≥2 to <18 years with rheumatoid-factor–positive or –negative polyarthritis, persistent or extended oligoarthritis, or systemic arthritis were enrolled into this prospective, observational, multicenter standard-of-care registry. Eligible patients were newly or recently prescribed (≤6 months) an nsNSAID or celecoxib. Enrolled patients were followed to the end of the study, whether they remained on the original NSAID, switched, or discontinued therapy altogether. All adverse events (AEs) regardless of severity were captured in the database.

**Results:**

A total of 274 patients (nsNSAID, n = 219; celecoxib, n = 55) were observed for 410 patient-years of observation. Naproxen, meloxicam, and nabumetone were the most frequently used nsNSAIDs. At baseline, the celecoxib group was older, had a numerically longer median time since diagnosis, and a numerically higher proportion of patients with a history of gastrointestinal-related NSAID intolerance. AEs reported were those frequently observed with NSAID treatment and were similar across groups (nsNSAIDs: 52.0%; celecoxib: 52.9%). Twelve unique patients experienced a total of 18 serious AEs; the most frequent were infections, and none was attributed to NSAID use.

**Conclusions:**

The safety profile of celecoxib and nsNSAIDs appears similar overall. The results from this registry, ongoing pharmacovigilance, and the phase 3 trial that led to the approval of celecoxib for children with JIA provide evidence that the benefit-risk for celecoxib treatment in JIA remains positive.

**Trial registration:**

ClinicalTrials.gov identifier NCT00688545.

## Background

Even in this age of targeted biologic therapies such as tumor necrosis factor-α inhibitors, nonsteroidal anti-inflammatory drugs (NSAIDs) are integral to the treatment of juvenile idiopathic arthritis (JIA). NSAIDs may be the only therapy required for symptom control in some patients with JIA, and when used as an adjunct to a disease-modifying antirheumatic drug (DMARD), clinical responses to NSAID therapy can be quite robust [1-6].

NSAIDs that inhibit both cyclooxygenase (COX)-1 and -2 enzymes (ie, nonselective or nsNSAIDs) are used in a large majority of patients with JIA, although few NSAIDs have been approved for this indication by regulatory agencies. As of 2005, the only nsNSAIDs approved for the treatment of JIA by the US Food and Drug Administration (FDA) were naproxen, ibuprofen, tolmetin, oxaprozin, and meloxicam. Of the COX-2–selective NSAIDs, rofecoxib (Merck) was approved for JIA in 2004; in December 2006 the FDA approved celecoxib (Pfizer) to treat the signs and symptoms of juvenile rheumatoid arthritis (JRA) in children aged 2–17 years. This approval was based on a double-blind randomized controlled study that compared celecoxib with naproxen in 242 patients over a 12-week period, with a 12-week open-label extension of 202 patients, contributing a total of 100 patient-years (PY) of exposure for celecoxib. The study demonstrated that the efficacy of celecoxib is noninferior to naproxen, with similar safety and tolerability profiles [3].

In late 2004, rofecoxib was voluntarily withdrawn globally due to cardiovascular (CV) safety concerns in adults. In 2005, another selective NSAID, valdecoxib (Pfizer), was also voluntarily withdrawn due to an unfavorable risk profile in adults, which in part related to the occurrence of severe cutaneous adverse reactions. Studies in adults have suggested that users of any NSAID may be at increased risk of CV outcomes, independent of the degree of selectivity of COX-2 inhibition [7]. The FDA reviewed the available data for all NSAIDs in 2005 and determined that celecoxib’s benefits outweighed its risks for appropriate patients.

While children have a very low risk of CV thromboembolic and serious gastrointestinal (GI) events, many patients with JIA will enter adulthood with arthritis and may require NSAID therapy for prolonged periods. Knowledge regarding adverse effects and long-term sequelae of treating children with NSAIDs is limited. The development program for celecoxib was similar to those for other NSAIDs approved for JIA; none of these programs were sufficiently large (median size, 59 patients) nor exposed patients for a long duration (median duration, 12 weeks) to exclude rare or latent effects of treatment.

To address the need for longer-term data from routine clinical practice regarding the safety of these medications in JIA patients and to fulfill a postapproval commitment to the FDA, we conducted the *S*afety in *I*diopathic Arthritis: *N*SAIDs and *C*elebrex *E*valuation *R*egistry (SINCERE™; ClinicalTrials.gov identifier #NCT00688545).

SINCERE was a phase 4 observational multicenter registry designed to collect longer-term safety data and measures of development (defined as growth velocity and pubertal maturation) from JIA patients in the United States who were prescribed celecoxib or nsNSAIDs. In consultation with the FDA, the study ended early due to difficulties in celecoxib patient recruitment as a result of an evolving JIA treatment paradigm that shortened the recommended duration of NSAID use, and the very low incidence rate of observed serious adverse events (SAEs).

## Methods

This was a multicenter, single-country (United States), prospective, observational registry of children and adolescents aged ≥2 to <18 years treated with celecoxib or nsNSAIDs by the treating rheumatologist for the signs and symptoms of select categories of JIA conducted from 2009–2012. Categories included persistent or extended oligoarthritis, rheumatoid-factor–positive or –negative polyarthritis, or systemic arthritis (without presence of extra-articular features for ≥6 months prior to enrollment) [8]. Enthesitis-related arthritis and psoriatic arthritis subtypes of JIA were excluded from the study population since celecoxib was approved under the older JRA indication. Given the expected recruitment difficulties in this rare disease and the treatment paradigm in which a child’s general pediatrician often initially prescribes an NSAID while waiting for further evaluation and, if necessary, referral to a pediatric rheumatologist, we could not restrict the study population to a true inception or new user design. Patients were eligible for participation if they received a prescription for celecoxib or an nsNSAID not more than 6 months prior (a quasi-inception cohort), although previous cumulative exposure to NSAIDs could exceed 6 months. Enrollment was subsequent to the rheumatologist’s decision to initiate or change an NSAID.

Registry assessments were performed at baseline, at approximately months 4, 8, and 12, and twice yearly thereafter. The minimum requested follow-up in the study was 2 years, with a target total sample size of 400 patients, 200 each in the celecoxib and nsNSAID arms.

Data collected included safety information, defined as all adverse events (AEs) and developmental outcomes (height, weight, and age of menarche if appropriate). The Gastrointestinal Scale for Kids (GISSK) instrument [9] served to assess GI tolerability at baseline and to screen for potential GI AEs during follow-up.

The following additional assessments were conducted: the Childhood Health Assessment Questionnaire [10] at baseline; the rheumatologic joint assessment at the initial and final visits, the physician’s global assessment of disease activity, and the parent assessment of overall well-being (both measured on an 11-point ordinal scale ranging from 0–10) at each visit.

### Drug information and supplies

The choice of study NSAID (celecoxib or nsNSAID), dosage, frequency, and any adjustments thereof were at the treating rheumatologist’s discretion. NSAID switches and discontinuations were recorded, and patients remained under follow-up, even if no longer using any NSAID.

### Concomitant medications

Concomitant DMARDs or biologics, or changes in these medications, were permissible as per the clinical judgment of the treating physician. Concurrent treatment with >1 NSAID was not allowed, though occasional use of a second nsNSAID or acetaminophen for pain or antipyresis was permitted.

### JIA diagnosis change

If a patient’s new diagnosis was another JIA category (eg, psoriatic arthritis or enthesitis-related arthritis), they remained in the study; the patient was withdrawn if the new diagnosis was another rheumatic or nonrheumatic disease (eg, lupus, fibromyalgia, or cancer).

### AE reporting

An AE was defined as any untoward medical occurrence in a study patient as assessed by the treating physician. An SAE was defined as any AE that resulted in death, was life-threatening, required hospitalization or prolongation of existing hospitalization, resulted in persistent or significant disability, or resulted in congenital anomaly. Pregnancy in a patient or patient’s partner was to be reported as an SAE, even if no AE occurred in the mother or child. The reporting period for all AEs extended from the time the patient provided informed consent through and including 28 calendar days after the last study visit.

### Growth velocity and pubertal maturation

Growth (height and weight) was assessed via chart abstraction for the year prior to enrollment and at each study encounter throughout the registry. Standard age-appropriate growth charts from the US Centers for Disease Control and Prevention [11] were utilized to monitor for important deviations from the norm. Age of menarche was recorded at enrollment or at later study encounter.

### Outcomes of interest

To address concerns about GI and CV safety with NSAIDs, several events of special interest were defined a priori: *GI events* consisted of ulcers or complications thereof; *CV/cardiorenal events* comprised thromboembolic events or new-onset/worsening hypertension; and *new-onset or worsening hypertension* could be identified either as an AE or via a change in antihypertensive drug use such as initiation or an increase in dose. A post hoc *composite GI event* category was developed to quantify GI tolerability events, namely nausea/vomiting, abdominal pain, and all other GI events.

### Confounder and other assessments

The following factors were collected: demographics (sex, race, age); targeted medical history, with particular attention to GI risk factors; previous history of GI bleeds; NSAID intolerability and other drug-related adverse reactions; presence of hypertension, prior thromboembolic disease, substantial changes in body weight, or other CV risk factors; medications and dosage, and reason for therapy switches or terminations; concomitant medications; and early withdrawal from the registry, including reason.

### Study ethics

The study protocol, informed consent, and assent documentation were reviewed and approved by the institutional review board(s) at each investigational center. The study was conducted in accordance with the Declaration of Helsinki, International Conference of Harmonisation Good Clinical Practice as appropriate, Good Pharmacoepidemiology Practices [12], and local regulatory requirements and laws. The parent or legal guardian of each eligible patient signed informed consent to participate in the registry and a medical release form, and children signed assent as required by the institutional review board(s).

### Treatment classification

Patients who switched NSAID treatments during the study were included in the treatment group that the patient was in at the time the data were observed. Changing from celecoxib to an nsNSAID (or vice versa) was considered a treatment switch, while changing from one nsNSAID to another was not.

Each participant’s total exposure time to each medication was calculated for the full study, even if patients switched medications. AEs were grouped in the main analysis according to the NSAID (celecoxib or nsNSAID) utilized at the time of the event, regardless of the initial NSAID treatment at enrollment. The safety analysis set, which was used for all analyses, consisted of all enrolled patients who were prescribed at least 1 dose of any NSAID, regardless of whether it was celecoxib or a nsNSAID.

### Statistical hypotheses and analyses

No a priori hypotheses were formulated for the analysis as the primary intent of the registry was to collect broad longer term safety information within this cohort of NSAID users with JIA. No formal power analyses were conducted because no comparative analyses were performed.

Due to small sample size, incidence rate ratios were not calculated, although incidence rates and 95% confidence intervals (CIs) around the incidence rates were calculated according to the method of Sahai and Khurshid [13] as adapted for CIs around individual rates. Only descriptive comparisons of baseline characteristics were performed for the study.

Kaplan-Meier analysis was used to display cumulative exposure time on initial NSAID treatment. SAS software (version 9.1, SAS Institute Inc, Cary, NC) was used to perform all analyses.

## Results

Sixteen Pediatric Rheumatology Collaborative Study Group member sites enrolled 274 patients from April 2009 until January 2012.

### Baseline characteristics

A total of 219 (80%) patients received an nsNSAID as initial treatment and 55 (20%) received celecoxib (Table 1). The celecoxib group had a numerically higher mean age than the nsNSAID group (10.0 vs. 8.2 years), with a relatively lower proportion in the youngest age group aged 2–5 years (14.5% vs. 35.2%) and a relatively greater proportion in the oldest adolescent group aged 16–18 years (14.5% vs. 5.0%). Patients in the nsNSAID group had a shorter median duration of disease by almost 8 months. Similar proportions of celecoxib (9.1%) and nsNSAID (11.9%) users changed JIA diagnoses over the course of the study.

**Table 1:** Patient demographics and other baseline characteristics

	**nsNSAID (n = 219)**	**Celecoxib (n = 55)**
Age, years, mean (SD)	8.2 (4.45)	10.0 (4.09)
Sex, n (%)		
Male	54 (24.7)	8 (14.5)
Female	165 (75.3)	47 (85.5)
Race, n (%)		
White	193 (88.1)	49 (89.1)
Black	16 (7.3)	5 (9.1)
Asian	6 (2.7)	0
Other	4 (1.8)	1 (1.8)
Ethnicity, n (%)		
Hispanic or Latino	19 (8.7)	2 (3.6)
Non-Hispanic or Latino	200 (91.3)	53 (96.4)
Body mass index categories, n (%)		
Underweight	10 (4.6)	6 (10.9)
Healthy weight	143 (65.3)	36 (65.5)
Overweight	38 (17.4)	8 (14.5)
Obese	20 (9.1)	4 (7.3)
Missing	8 (3.7)	1 (1.8)
Months since JIA diagnosis, median (range)	4.25 (0–163.0)	12.10 (0.5–173.5)
JIA category, n (%)		
Systemic	3 (1.4)	0
Persistent oligoarticular	119 (54.3)	27 (49.1)
Extended oligoarticular	12 (5.5)	2 (3.6)
Poly RF (–)	70 (32.0)	23 (41.8)
Poly RF (+)	14 (6.4)	3 (5.5)
Missing	1 (0.5)	0
Patients that have discontinued ≥1 NSAID in prior year,^a^ n (%)	114 (52.1)	46 (83.6)
AE	16 (7.3)	25 (45.5)
Other reasons	64 (29.2)	15 (27.3)
Lack of efficacy	40 (18.3)	10 (18.2)
Both AE/lack of efficacy	3 (1.4)	4 (7.3)
Rheumatologic joint assessment, mean (SD)		
Number of joints with active arthritis^b^	2.9 (4.1)	3.4 (5.4)
Number of joints with swelling	2.7 (4.0)	3.1 (5.1)
Number of joints with pain on motion/tenderness	1.7 (3.6)	2.5 (4.3)
Number of joints with loss of motion	1.7 (3.0)	1.9 (3.2)
Childhood health assessment questionnaire, median (range)		
Functioning (in past week)^c^	0.35 (0–2.5)	0.44 (0–2.5)
Pain^d^	0.45 (0–3.0)	0.66 (0–2.5)
Global evaluation^e^	14.00 (0–87.0)	16.50 (0–97.0)
Physician’s Global Assessment of Disease Activity,^f^ mean (SD)	2.9 (2.2)	2.6 (2.4)
Parent/subject assessment of overall well being,^g^ mean (SD)	2.8 (2.5)	3.2 (2.6)

^a^Patients discontinuing more than 1 NSAID for the same reason are only counted once in the overall summary; however, if a patient discontinued an NSAID for more than 1 reason, the patient contributes to the summary of each of those reasons.

^b^Active arthritis is defined as swelling of the joint or, if no swelling is present, loss of motion accompanied by pain on motion or tenderness.

^c^Functioning can range from 0–3, where higher scores indicate more impairment.

^d^Pain can range from 0–3, where 0 = no pain and 3 = very severe pain.

^e^Global evaluation can range from 0–100, where 0 = very well and 100 = very poor.

^f^Physician’s Global Assessment can range from 0–10, where 0 = not active and 10 = very active.

^g^Parent/subject assessment of overall well-being can range from 0–10, where 0 = very well and 10 = very poorly.

*Abbreviations AE*: Adverse event, *JIA* Juvenile idiopathic arthritis, *nsNSAID* Nonselective nonsteroidal anti-inflammatory drug, *RF* Rheumatoid factor.

The nsNSAID and celecoxib groups were similar at baseline for the rheumatologic joint assessment, physician’s global assessment of disease activity, and parent/subject assessment of overall well-being (Table 1). Median weight and height were numerically greater in the celecoxib group than in the nsNSAID group, which is consistent with the age difference. The celecoxib group also had numerically worse results than the nsNSAID group at baseline for all components of the Childhood Health Assessment Questionnaire and GISSK. Differences between groups were greatest for the GISSK’s mean severity score (celecoxib 21.9, nsNSAID 12.0) and percentages of patients with specific problems, mainly lower stomach pain (celecoxib 29.1%, other NSAID 15.5%) and nausea (celecoxib 25.5%, nsNSAID 14.6%). Correspondingly, the celecoxib group had a numerically higher proportion of patients with a history of GI-related NSAID intolerance consistent with other baseline assessments of GI conditions.

### Drug exposure and PY of observation

**Figure 1:**
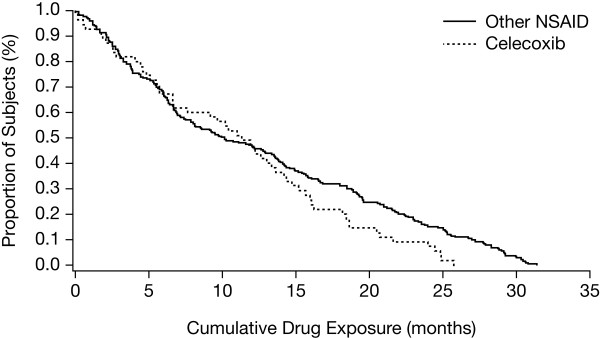
Kaplan-Meier plot comparing cumulative time on baseline study drug.

Mean duration of baseline treatment was similar for the nsNSAID and celecoxib groups (12.5 and 11.2 months, respectively). Exposure to baseline treatment was 228.1 PY for nsNSAIDs and 51.3 PY for celecoxib. Analysis of cumulative time of study drug exposure during baseline treatment after study enrollment did not indicate substantial differences between groups in the distribution of time on treatment (Figure 1). In addition, the proportion of patients lost to follow-up over the course of the study was small and equal in both groups (1.8%, representing 4 patients and 1 patient in the nsNSAID and celecoxib groups, respectively).

Naproxen (47.8%), meloxicam (17.9%), and nabumetone (6.6%) were the most frequently prescribed nsNSAIDs. At the time of study termination, 79 patients (28.8% of total) had discontinued NSAID treatment, 11 initially treated with celecoxib and 68 initially treated with another NSAID; furthermore, 67.6% of those on nsNSAIDs and 70.9% of those on celecoxib were receiving concomitant medications for JIA (DMARDs, biologics, corticosteroids, and analgesics).

NSAID treatments were switched at least once during the study for 19 patients (6.9% of the total population). There were 225 patients in the nsNSAID group (219 at baseline and 6 switched from celecoxib) and 68 in the celecoxib group (55 at baseline and 13 switched from nsNSAIDs).

The mean observation time was 14.3 months in the nsNSAID group and 12.0 months in the celecoxib group, and 11.3 months for patients whose NSAID therapy was discontinued ≥29 days after last dose of study medication; the medians were generally similar. The study observed patients for a total of 410.2 PY; individual study medication contributions can be found in Table 2. Mean total follow-up time for all patients regardless of treatment group and off-NSAID observation was 18.0 months.

**Table 2:** AEs of special interest and composite gastrointestinal AEs by current treatment group (safety analysis set)

**Variable/event type/AE**^**a**^	**nsNSAID (n = 225**^**b**^**)**	**Celecoxib (n = 68**^**b**^**)**	**Off NSAID**^**c **^**(n = 79)**
**Incidence, n (%)**			
Patients with at least 1 AE of special interest	2 (0.9)	0	2 (2.5)
GI	0	0	1 (1.3)
GI ulcer	0	0	0
Complications of GI ulcer	0	0	1 (1.3)
Cardiovascular/cardiorenal	2 (0.9)	0	1 (1.3)
Thromboembolic events	0	0	0
New-onset or worsening hypertension	2 (0.9)	0	1 (1.3)
Patients with at least 1 GI composite event	73 (32.4)	23 (33.8)	17 (21.5)
Abdominal pain	39 (17.3)	12 (17.6)	6 (7.6)
Nausea/vomiting	31 (13.8)	13 (19.1)	8 (10.1)
All other GI	44 (19.6)	11 (16.2)	11 (13.9)
**Incidence rate (per 100 person-years), (95% CI)**			
Total person-years	267.6	68.0	74.6
Incidence rate of events of special interest	0.7 (0–1.8)	0	2.7 (0–6.4)
GI	0	0	1.3 (0–4.0)
Gl ulcer	0	0	0
Complications of GI ulcer	0	0	1.3 (0–4.0)
Cardiovascular/cardiorenal	0.7 (0–1.8)	0	1.3 (0–4.0)
Thromboembolic events	0	0	0
New-onset or worsening hypertension	0.7 (0–1.8)	0	1.3 (0–4.0)
Incidence rate of GI composite events	27.3 (21.0–33.5)	33.8 (20.0–47.6)	22.8 (12.0–33.6)
Abdominal pain	14.6 (10.0–19.1)	17.6 (7.7–27.6)	8.0 (1.6–14.5)
Nausea/vomiting	11.6 (7.5–15.7)	19.1 (8.7–29.5)	10.7 (3.3–18.2)
All other GI	16.4 (11.6–21.3)	16.2 (6.6–25.7)	14.7 (6.0–23.5)

^a^Patients are counted only once in each AE of interest category, and only once in each event type category.

^b^Patients will appear in both treatment groups if they switched treatments.

^c^Off NSAID is defined as 29 or more days after last dose of final study medication.

*Abbreviations: AE* Adverse event, *CI* Confidence interval *GI* Gastrointestinal, *NSAID* Nonsteroidal anti-inflammatory drug.

### Safety

As summarized in Table 3, all-causality AEs were reported for similar percentages of patients during nsNSAID and celecoxib treatment (52.0% vs. 52.9%), and during off-NSAID observation (46.8%); the corresponding incidence rates were 43.7, 52.9, and 49.6 per 100 PYs and the mean duration of observation was similar among these groups. Furthermore, the rates of temporary discontinuation were low (<10.3%) but numerically more common in the celecoxib group.

**Table 3:** Summary of AEs by current treatment group

**AE category**	**nsNSAID (n = 225**^**a**^**) n (%)**	**Celecoxib (n = 68**^**a**^**) n (%)**	**Off NSAID**^**b **^**(n = 79) n (%)**
Patients with at least 1 AE	117 (52.0)	36 (52.9)	37 (46.8)
Patients with at least 1 AE causing temporary discontinuation	14 (6.2)	7 (10.3)	2 (2.5)
Patients with at least 1 serious AE	9 (4.0)	2 (2.9)	3 (3.8)
Patients with at least 1 AE of special interest	2 (0.9)	0	2 (2.5)
Patients with at least 1 GI composite event^c^	73 (32.4)	23 (33.8)	17 (21.5)

^a^Patients who switch treatments contribute to both treatment groups.

^b^Off NSAID is defined as 29 or more days after last dose of final study medication.

^c^Nausea/vomiting, abdominal pain, or all other GI.

*Abbreviations: AE* Adverse event, *GI* Gastrointestinal, *nsNSAID* Nonselective nonsteroidal anti-inflammatory drug.

Percentages of patients with any AE considered treatment-related were similar with nsNSAID and celecoxib treatment (21.3% vs. 19.1%; Table 4). GI disorders were the most frequently reported type of treatment-related AE. Other types of treatment-related AEs were reported for ≤2.9% patients during either study treatment; 7.6% of patients in the off-treatment group reported an AE considered treatment-related.

**Table 4:** AEs considered treatment-related, by current treatment group

**MedDRA system organ class/preferred term**^**a**^	**nsNSAID (n = 225**^**b**^**) n (%)**	**Celecoxib (n = 68**^**b**^**) n (%)**	**Off NSAID**^**c **^**(n = 79) n (%)**
Patients with at least 1 treatment-related AE	48 (21.3)	13 (19.1)	6 (7.6)
GI disorders (as shown below)	36 (16.0)	10 (14.7)	3 (3.8)
Abdominal pain	17 (7.6)	5 (7.4)	0
Nausea	8 (3.6)	3 (4.4)	1 (1.3)
Diarrhea	6 (2.7)	2 (2.9)	0
Dyspepsia	5 (2.2)	2 (2.9)	1 (1.3)
Gastroesophageal reflux disease	6 (2.7)	0	0
Constipation	4 (1.8)	0	1 (1.3)
Abdominal pain lower	3 (1.3)	0	0
Abdominal pain upper	1 (0.4)	2 (2.9)	0
Vomiting	0	1 (1.5)	1 (1.3)
Abdominal distension	1 (0.4)	0	0
Feces discolored	0	1 (1.5)	0
Gastritis	1 (0.4)	0	0
General disorders and administration site conditions	3 (1.3)	0	0
Immune system disorders (drug hypersensitivity)	1 (0.4)	0	0
Infections and infestations	1 (0.4)	0	1 (1.3)
Injury, poisoning, and procedural complications	4 (1.8)	0	0
Investigations	2 (0.9)	0	0
Blood pressure increased	1 (0.4)	0	0
Blood urea increased	1 (0.4)	0	0
Weight decreased	1 (0.4)	0	0
Metabolism and nutrition disorders	5 (2.2)	1 (1.5)	2 (2.5)
Musculoskeletal and connective tissue disorders	1 (0.4)	0	0
Nervous system disorders	6 (2.7)	0	0
Renal and urinary disorders	0	2 (2.9)	0
Reproductive system and breast disorders	0	1 (1.5)	0
Skin and subcutaneous tissue disorders	4 (1.8)	0	2 (2.5)
Vascular disorders	1 (0.4)	0	0

^a^Patients are counted only once in each preferred term category, and only once in each system organ class category.

^b^Patients appear in both treatment groups if they switched treatments.

^c^Off NSAID is defined as 29 or more days after last dose of final study medication.

*Abbreviations: AE* Adverse event, *MedDRA* Medical Dictionary for Regulatory Activities, *GI* Gastrointestinal, *NSAID* Nonsteroidal anti-inflammatory drug.

SAEs were reported for 9 patients (4.0%) during nsNSAID treatment, 2 events (2.9%) during celecoxib treatment, and 3 events (3.8%) during off-NSAID observation (Table 5). There were 12 unique patients with SAEs because 2 patients had SAEs during more than 1 treatment/observation period and were counted twice for this analysis. Infections were the most frequently reported type of SAE. In an analysis by baseline treatment group, the SAE rate/100 PY was 3.0 (95% CI, 1.1–4.8) for patients started on nsNSAID and 2.6 (95% CI, 0.0–6.3) for patients started on celecoxib. No SAE was thought to be attributable to the study medications.

**Table 5:** Serious adverse events by current NSAID treatment

**Patient ID**	**Current medication**	**Onset-resolution (day**^**a**^**)**	**Reported description**	**Outcome**	**Action taken (study medication)**	**Causality**
A	nsNSAID	729–733	Cellulitis	Resolved	No action taken	Other: spider bite
B	nsNSAID	478–481	Benign fibrohistiocytic lesions with giant cells	Resolved	No action taken	Other: idiopathic hip pain
C	nsNSAID	303–307	Appendicitis	Resolved	No action taken	Other: appendicitis
D	nsNSAID	27–N/A	Unintended pregnancy	Unknown	Permanently discontinued	Other: pregnancy
E	nsNSAID	33–39	Meningitis	Resolved with sequelae	Permanently discontinued	Other illness: infection—probably viral
F	nsNSAID	160–167	Fever and neutropenia	Resolved with sequelae	Stopped temporarily	Other illness: mycoplasma infection
F	nsNSAID	161–167	Agranulocytosis	Resolved with sequelae	No action taken	Other illness: mycoplasma infection
G	nsNSAID	236–288	Infection musculoskeletal septic arthritis	Resolved	Stopped temporarily	Other: unknown
H	nsNSAID	205–N/A	Onset of systemic JIA	Still present	Permanently discontinued	Other: systemic JIA
I	nsNSAID	93–149	Right lower quadrant abdominal pain	Resolved	Stopped temporarily	Other: unknown possible subclinical appendicitis but pathology negative
I	Celecoxib	138–149	Phlegmon	Resolved	Stopped temporarily	Other: postsurgical (appendectomy) complication
I	Celecoxib	659–N/A	Transformed migraine	Still present	Stopped temporarily	Other illness: likely related to past medical history of migraines
J	Celecoxib	82–246	Exacerbation of disease	Resolved	No action taken	Disease under study
J	Celecoxib	82–132	Fever	Resolved	No action taken	Disease under study
K	Off NSAID	69–N/A	Primary sclerosing cholangitis	Still present	Permanently discontinued	Other illness: primary sclerosing cholangitis
G	Off NSAID	452–N/A	Septic arthritis	Still present	No action taken	Other illness: septic arthritis of unknown etiology
G	Off NSAID	471–512	Acute tubular necrosis	Resolved	Permanently discontinued	Other: vancomycin
L	Off NSAID	424–429	Febrile neutropenia	Resolved	No action taken	Other illness: infection questionable Rocky Mountain spotted fever

^a^Relative to the start of treatment.

*Abbreviations: JIA* Juvenile idiopathic arthritis, *N/A* Not available, *nsNSAID* Nonselective nonsteroidal anti-inflammatory drug.

Only 2 patients had AEs of special interest (new-onset or worsening hypertension for both) during study treatment (nsNSAID in both cases; Table 2). Two additional AEs of special interest (complications of GI ulcer and new-onset or worsening hypertension) occurred at least 29 days after the study medication (initially treated with celecoxib and nsNSAID, respectively) was stopped.

The incidence rates/100 PY for AEs of special interest during nsNSAID treatment and off-NSAID observation were small (0.7 [95% CI, 0–1.8] and 2.7 [95% CI, 0–6.4], respectively). Percentages of patients with GI composite AEs and incidence rates/100 PY of these AEs were generally similar for the nsNSAID and celecoxib groups.

## Discussion

Celecoxib had safety outcomes similar to nsNSAIDs in a total study population of 274 JIA patients followed for a mean duration of approximately 18 months, corresponding to a total of 410 PY of observation. These data add considerably to the body of knowledge regarding NSAID treatment of JIA. The pivotal celecoxib JIA trial [3], one of the largest randomized trials of NSAIDs in JIA, followed patients for 100 PY.

The analysis of AEs reported during study treatment generally showed a similar incidence of AEs across groups, and were those that are frequently observed with NSAID treatment (eg, GI-related). Overall, the study results did not indicate a difference in safety profile between celecoxib and other nsNSAIDs. However, the analysis is limited by the study’s early termination and reduced sample size as a result. The study would not be able to adequately address the original research question regarding the *long-term* safety of celecoxib in JIA patients due to A) difficulties in celecoxib patient recruitment (despite numerous attempts, only 20% of the 50% targeted was ever achieved); B) the change in the JIA treatment paradigm that emphasizes NSAID use for relatively short periods of time [14,15], and C) the very low incidence rate of observed SAEs. Furthermore, other accumulating safety data from sources such as the pharmacovigilance database and a simultaneously conducted active surveillance program [16] did not suggest any new signals in the JIA population.

The observational design demonstrated the expected differences between prescribed groups in baseline characteristics and baseline GI risk, indicative of confounding by indication. For example, on study entry, compared with nsNSAID users, the celecoxib users had evidence of more severe JIA disease and longer disease duration, reflecting the use of celecoxib as a second-line (or more) NSAID therapy in JIA. As expected, a history of GI problems and NSAID intolerance was more prevalent in celecoxib users, suggesting that celecoxib was preferentially prescribed by physicians to patients with a demonstrated history of nsNSAID intolerance. Due to the very limited sample size in the celecoxib group, methods such as propensity scores or multivariable regression could not be adequately addressed in analyses. However, based on the above, the study results should provide a conservative estimate of the safety of celecoxib compared with nsNSAIDs. It should be noted that meloxicam, utilized by 18% of the cohort, exhibits some COX-2–preferential selectivity in some assays; in our analyses, we grouped it with the nsNSAIDs, as the FDA considers it a nonselective NSAID.

SINCERE had numerous strengths. The broad inclusion criteria reflect a more generalizable JIA population than typically participate in randomized clinical trials for drug registration. The demographics and clinical characteristics of the study participants mirror the treated JIA population in the literature [17], suggesting the study is representative of JIA patients in general. Furthermore, the observational design—with treatment assignment decided by the patient’s treating rheumatologist—enabled assessment of actual clinical practice and the “real-life” safety profile of NSAIDs used for this disease. The study was designed a priori to capture detailed information on GI and CV risks and potential confounders to address potential confounding by indication. Despite the early end, this study had very little loss to follow-up for a longer-term observational study (1.8% in each group), had very little switching across NSAID groups (<7%), and recorded much longer mean follow-up time (18 months) than published randomized controlled trials of NSAIDs, which typically had 3–6 months’ follow up.

There are several limitations of the registry. Early termination of the study and unexpectedly low celecoxib use in the patient population did not allow for analyses of growth velocity and pubertal maturation or formal comparative safety analyses. Recruitment difficulties causing early termination or substantial extensions of enrollment in pediatric studies are a well-known challenge [18-20]. Furthermore, the use of biologics approximately doubled over the course of the study (data not shown) and is consistent with the increasing prevalence of biologic use with associated decline in NSAID use, as demonstrated in a drug utilization study from a large US insurer [21]. The duration of study treatment was relatively short and thus longer-term or latent AEs could not be adequately detected, but this short duration likely reflects actual current clinical practice with NSAIDs [22] in the United States, as suggested by recent guidelines from the American College of Rheumatology on the treatment of JIA [14,15]. Extensive use of concomitant medications, especially biologics and DMARDs, was a complicating factor for attribution of observed AEs to study treatment. Selection bias may have occurred; a new-user (NSAID-naïve) design would have minimized this potential bias, but the quasi-inception cohort design was chosen to maximize generalizability and operational efficiency as the number of celecoxib users was anticipated to be substantially less than nsNSAID users. Lastly, as this study was conducted exclusively in the United States, it is unknown whether other regions would have the same findings.

## Conclusions

The safety profile of celecoxib in JIA appears similar overall to that of nsNSAIDs, with few appreciable differences observed in this study. The total study population of 274 patients observed for a total of 410 PY is the largest prospectively followed JIA NSAID cohort to date, and adds considerably to the safety experience of NSAID treatment for JIA. Based on the findings of this study and the low number of SAEs observed overall, no new safety concerns were identified. These registry data, the ongoing pharmacovigilance, and the results of the phase 3 trial that led to the approval of celecoxib for children with JIA further support a positive benefit-risk balance for celecoxib in JIA.

## Abbreviations

CI: Confidence interval; COX: Cyclooxygenase; CV: Cardiovascular; DMARD: Disease-modifying antirheumatic drug; FDA: US Food and Drug Administration; GI: Gastrointestinal; JIA: Juvenile idiopathic arthritis; JRA: Juvenile rheumatoid arthritis; NSAIDs: Nonsteroidal anti-inflammatory drugs; nsNSAIDs: Nonselective NSAIDs; PY: Patient-years.

## Competing interests

RES and SA are employees and stockholders of Pfizer Inc. DJL has received support and honoraria from Centocor Inc., AstraZeneca, Wyeth Pharmaceuticals, Amgen, Bristol-Myers Squibb, Abbott Immunology Pharmaceuticals, Pfizer Inc., Regeneron, Hoffmann-LaRoche, Inc., Novartis Pharmaceutical Corporation, Forest Laboratories, Horizon Pharmaceuticals. BSG, PWM, and EHG have received support from Pfizer. MS has received honoraria from Abbott, Amgen, Genentech, Novartis, and Pfizer. All other co-authors have no disclosures.

## Authors’ contributions

RES conceived the study; RES, SA, EHG, DJL, BSG, and PWM participated in the design, scientific and operational oversight, and interpretation of the study, JPY performed data analysis and interpretation, and EHG and RES drafted the manuscript. All authors except RES, SA, JPY, and EHG contributed to data acquisition. All authors have read and approved the final manuscript.

## Authors’ information

Results of this study add further to the growing body of evidence, in a new population of patients, that the safety and tolerability of celecoxib are comparable to that of nonselective NSAIDs. The Pediatric Rheumatology Collaborative Study Group (PRCSG) was founded in 1973 and has more than 150 members at more than 90 academic clinical pediatric rheumatology centers in the United States, Canada, and Puerto Rico. The chief aim of the PRCSG is to conduct high-quality clinical trials of therapeutic agents in children with rheumatic diseases. Activities are overseen by an Advisory Council. DJL is the chairman, HIB is the scientific director, and EHG is the immediate past-senior scientist of the group.

DJL, HIB, JEW, PWM, BSG, ECC, LKJ, KBO, LP, DPG, KN, and MS are practicing pediatric rheumatologists at academic and/or children’s specialty hospitals and hold a number of senior academic and institutional leadership positions. EHG, RES, and SA are experienced pharmacoepidemiologists with additional expertise in conducting pediatric observational studies; RES is also the cofounder and co-chair of the International Society for Pharmacoepidemiology’s Special Interest Group in Pediatrics. JPY is a statistician who has worked in pharmaceutical clinical trials for >10 years.
